# The Importance of Particle Shape: Effect of Nonspherical Particles and Their Stabilized Pickering Emulsions on Immunization Efficacy

**DOI:** 10.1002/smsc.202400527

**Published:** 2025-04-20

**Authors:** Yanan Li, Jie Teng, Qiuting Chen, Qingze Fan, Hiroyuki Oku, Guanghui Ma, Jie Wu

**Affiliations:** ^1^ State Key Laboratory of Biochemical Engineering Institute of Process Engineering Chinese Academy of Sciences Beijing 100190 P. R. China; ^2^ Key Laboratory of Biopharmaceutical Preparation and Delivery Chinese Academy of Sciences Beijing 100190 P. R. China; ^3^ Division of Molecular Science Graduate School of Science & Engineering Gunma University Gunma 376‐8515 Japan; ^4^ Yantai Research Institute Harbin Engineering University Yantai Shandong 264006 P. R. China; ^5^ School of Biomedical Sciences and Engineering South China University of Technology Guangzhou Guangdong 510006 P. R. China; ^6^ Department of Pharmacy The Affiliated Hospital of Southwest Medical University Luzhou Sichuan 646000 P. R. China; ^7^ School of Chemistry and Chemical Engineering University of Chinese Academy of Sciences Beijing 100049 P. R. China

**Keywords:** cellular immunities, immune effects, nonspherical particles, pickering emulsion, vaccine adjuvants

## Abstract

Adjuvants based on spherical particles and stabilized Pickering emulsions represent a significant area of research in the adjuvant field. However, the immune effects of adjuvants containing nonspherical particles and their stabilized Pickering emulsions remain largely unexplored. Particle shape plays a critical role in influencing particle–cell interactions and antigen storage efficiency. In this study, it is aimed to synthesize nonspherical particles with diverse morphologies and sizes and successfully utilize one type to stabilize Pickering emulsions. In this research, their immunological effects are further evaluated by examining the activation of antigen‐presenting cells (APCs) and their impact on both cellular and humoral immunity. In these findings, it is demonstrated that nonspherical particles extend the in vivo residence time of vaccines and enhance APCs activation, thereby improving cellular immunity. Additionally, Pickering emulsions stabilized by nonspherical particles exhibit superior flexibility, higher antigen uptake by APCs, and more robust APCs activation compared to those stabilized by spherical particles. These advantages ultimately result in enhanced humoral and cellular immune responses.

## Graphical abstract

Poly (lactic‐co‐glycolic acid) is used to prepare nonspherical particles—macrofibers, macrothreads, and nanothreads (PNTs)—via one‐step solvent phase separation. PNT‐stabilized Pickering emulsions exhibit superior humoral and cellular immune enhancement over spherical particles and emulsions, highlighting the potential of nonspherical particles and their composite adjuvants in vaccine applications.

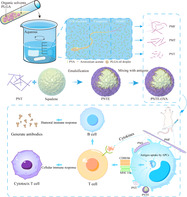

## 1 Introduction

In recent years, particle adjuvants have become a popular area of research,^[^ ^1^, ^2^ ^]^ with properties such as particle size, charge, and hydrophilicity affecting immune efficacy.^[^ ^3^ ^]^ Previous studies have mostly focused on spherical particles due to their formation, which is favored by the surface tension of the solutions.^[^ ^4^ ^]^ In addition, spherical particles have a large specific surface area and adjustable surface chemistry,^[^ ^5^ ^]^ making them effective in carrying antigens. However, recent studies have focused on the effects of different particle morphologies on immunity. For instance, rod‐shaped particles have been shown to be more advantageous for cellular uptake than spherical particles.^[^ ^6^ ^]^ The fact that particles are more favorable for cell uptake enhances antigen presentation, and the enhancement of immune cell activity is more beneficial for the improvement of immune effects.^[^ ^7^ ^]^ Despite these findings, what is often studied are single nonspherical particles. However, the immune effects of nonspherical particles combined with other formulations as adjuvants remain unclear. For example, a Pickering emulsion is a composite adjuvant whose oil phase is stabilized by particles.^[^ ^8^ ^]^

Pickering emulsions, a distinctive type of emulsion in which solid particles are dispersed in a liquid to create a stable system,^[^ ^9^ ^]^ have gained attention as biomimetic adjuvants because of their ability to mimic pathogens with high biosafety and antigen‐loading capacity.^[^ ^10^, ^11^, ^12^, ^13^, ^14^, ^15^ ^]^ However, existing research on Pickering emulsions as immune adjuvants has relied mainly on spherical particle‐stabilized Pickering emulsions. Recent studies have explored various nonspherical particle shapes, such as rods,^[^ ^16^ ^]^ ellipsoids,^[^ ^17^ ^]^ and platelets,^[^ ^18^ ^]^ demonstrating their unique advantages in specific Pickering emulsion applications.^[^ ^19^, ^20^ ^]^ For example, recent research indicates that rod‐shaped particles can achieve higher emulsion stability due to their ability to form a dense interfacial layer, thereby reducing coalescence.^[^ ^21^ ^]^ However, these studies often focus on the physicochemical properties of the emulsions and provide limited insights into their biological applications, particularly in the context of vaccine adjuvants.

To address these limitations, nonspherical particles have been prepared using poly(lactic‐co‐glycolic acid) (PLGA), a biodegradable polymer widely used in medicine.^[^ ^22^ ^]^ The preparation of these particles followed the principles of solvent phase separation and the influence of salt ions in solution on particle morphology after solidification.^[^ ^23^ ^]^ Based on this, the selection of particles was optimized, and the preparation conditions of the composite adjuvant were explored. A stable Pickering emulsion was successfully prepared from the smallest nonspherical particles. The immune effects of different sizes of nonspherical particles and spherical/nonspherical particle‐stabilized Pickering emulsions as vaccine adjuvants were evaluated.

## 2 Result and Discussion

### 2.1 Characterization of PLGAs with Different Size Aspect Ratios

**Figure 1:**
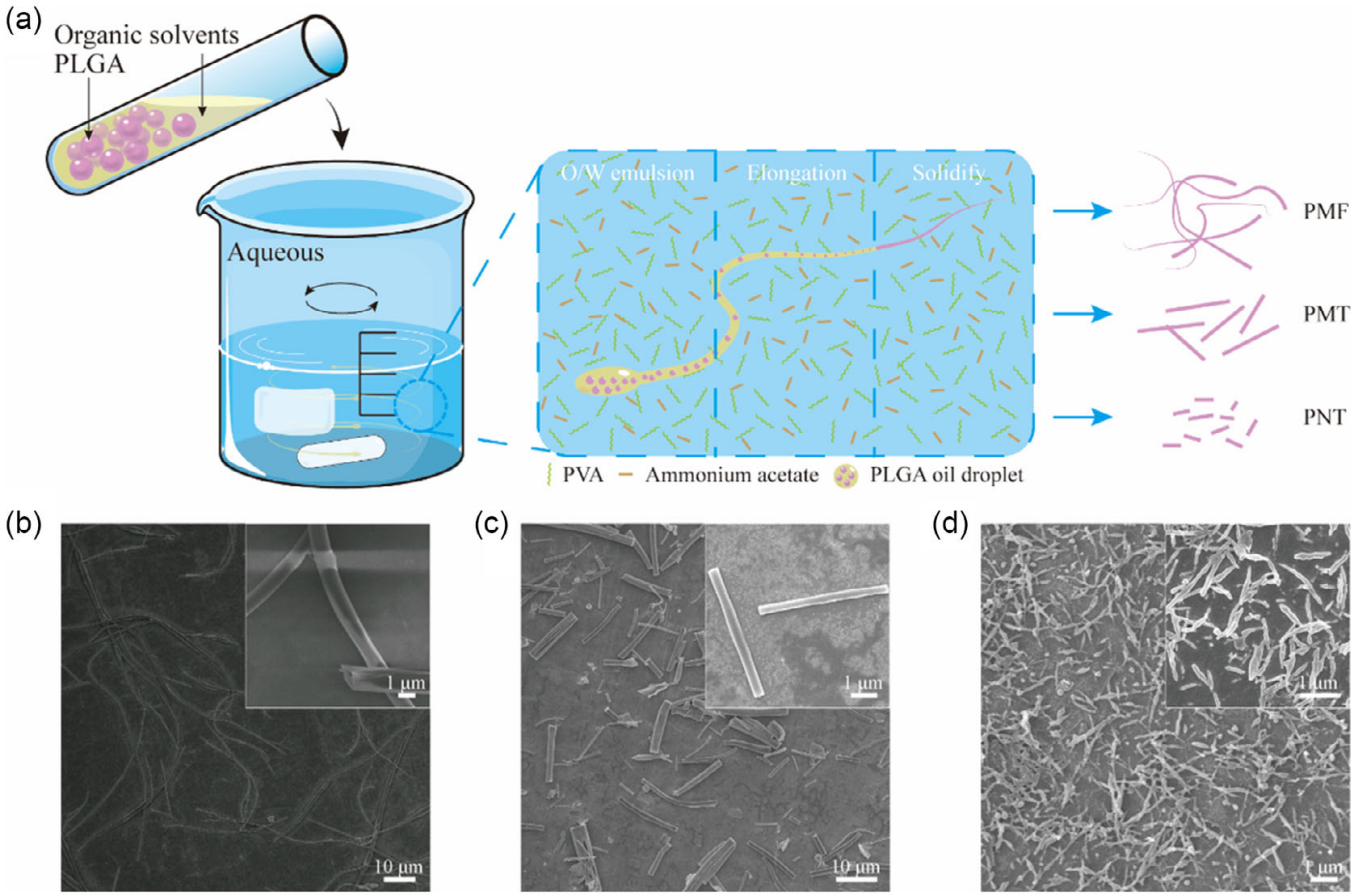
Characterization of PLGA particles with three different aspect ratios. a) Schematic diagram of the preparation process and SEM images of b) PMFs, c) PMTs, and d) PNTs.

Compared to spherical particles, the preparation of nonspherical particles often requires specific material selection, and the preparation process is complex and difficult to control in terms of shape and size, resulting in relatively low production efficiency.^[^ ^24^ ^]^ Previous studies have shown that the addition of salt ions to the aqueous phase during preparation can affect the morphology of PLGA after curing.^[^ ^23^ ^]^ In contrast to the preparation of spherical PLGA nanoparticles (PNPs), the process of preparing PLGA with different aspect ratios is illustrated in **Figure** **1**a. By adding salt ions to the external aqueous phase and controlling the phase separation rate of PLGA, the morphology of PLGA changes, resulting in the formation of PLGA microfibers (PMFs), PLGA microthreads (PMTs), and PLGA nanothreads (PNTs). The scanning electron microscope (SEM) images show the different aspect ratios of the three types of particles, and their lengths and diameters were analyzed using Nano Measure 1.2 software.

PMFs (Figure 1b) are presented as elongated filamentous microparticles, boasting a maximum length of 56.39 ± 5.49 μm (Figure S1a, Supporting Information) and a larger diameter of 1.23 ± 0.04 μm (Figure S1b, Supporting Information). PMTs (Figure 1c) adopt a rod‐shaped structure with a length of 9.49 ± 0.54 μm (Figure S1c, Supporting Information) and a diameter of 1.56 ± 0.03 μm (Figure S1d, Supporting Information), which was achieved through the sonication of the PMF. PNTs (Figure 1d), the smallest rodlike nanoparticles in scale among all the particles, exhibit a length of 763.90 ± 5.86 nm (Figure S1e, Supporting Information) and a diameter of 119.80 ± 2.51 nm (Figure S1f, Supporting Information).

### 2.2 Characterization of PNT‐Stabilized Pickering Emulsions

**Figure 2:**
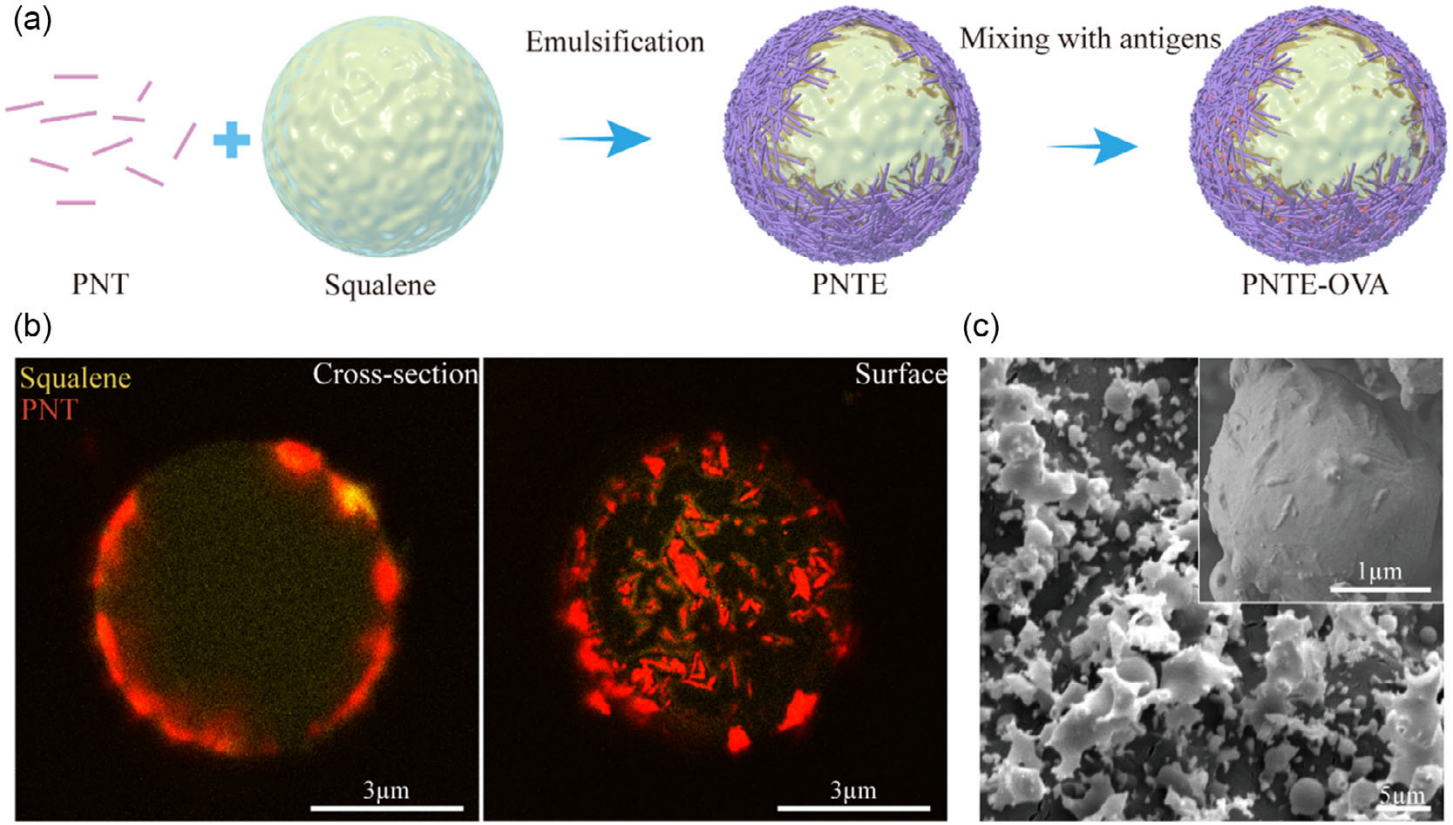
Characterization of PNT‐stabilized Pickering emulsions (PNTEs). a) Schematic diagram of preparation of PNTEs. b) CLSM images of the cross section and surface of PNTEs, squalene was labeled by Nile red (yellow) and PNTs were labeled by Cy5 (red). c) Cryo‐SEM image of PNTEs.

In previous studies, spherical particle‐stabilized Pickering emulsions had similar viscoelasticity, fluidity, and surface roughness to those of natural pathogens,^[^ ^10^ ^]^ whereas nonspherical particle‐stabilized Pickering emulsions as vaccine adjuvants have rarely been reported. To compare the differences between Pickering emulsions stabilized by spherical and nonspherical particles, Pickering emulsions stabilized by nonspherical particles were successfully prepared using PNTs. PMFs and PMTs failed to stabilize the emulsion due to their larger particle sizes, which led to emulsion aggregation and uneven particle distribution (Figure S2, Supporting Information), whereas PNTs had the characteristics of small particle size and high aspect ratio. The contact angle between PNTs and water was 75.1° (Figure S3, Supporting Information) similar to that of PNPs at 75.8°.^[^ ^25^ ^]^ This is mainly because the contact angle is related to the surface properties of the material rather than to the shape. According to Bancroft rules, hydrophilic particles can better stabilize O/W emulsions and form stable Pickering emulsions (**Figure** **2**a).^[^ ^26^ ^]^ The zeta potential of PNTs in water is −17.8 ± 2.3, which is due to the high concentration of carboxyl groups on their surfaces, preventing emulsion agglomeration and making it more stable. To observe the arrangement of nonspherical particles on the surface of the emulsion more easily, we shortened the ultrasonic time to 30s to prepare the Pickering emulsion with a larger particle size. Nonspherical particles were observed on the surface of the PNT‐stabilized Pickering emulsions **(**PNTEs), as shown in the confocal laser scanning microscopy (CLSM) (Figure 2b) and cryo‐SEM (Figure 2c) images. The particles on the surface of the PNTEs exhibited discontinuities with interstitial spaces that favor encapsulation.

**Figure 3:**
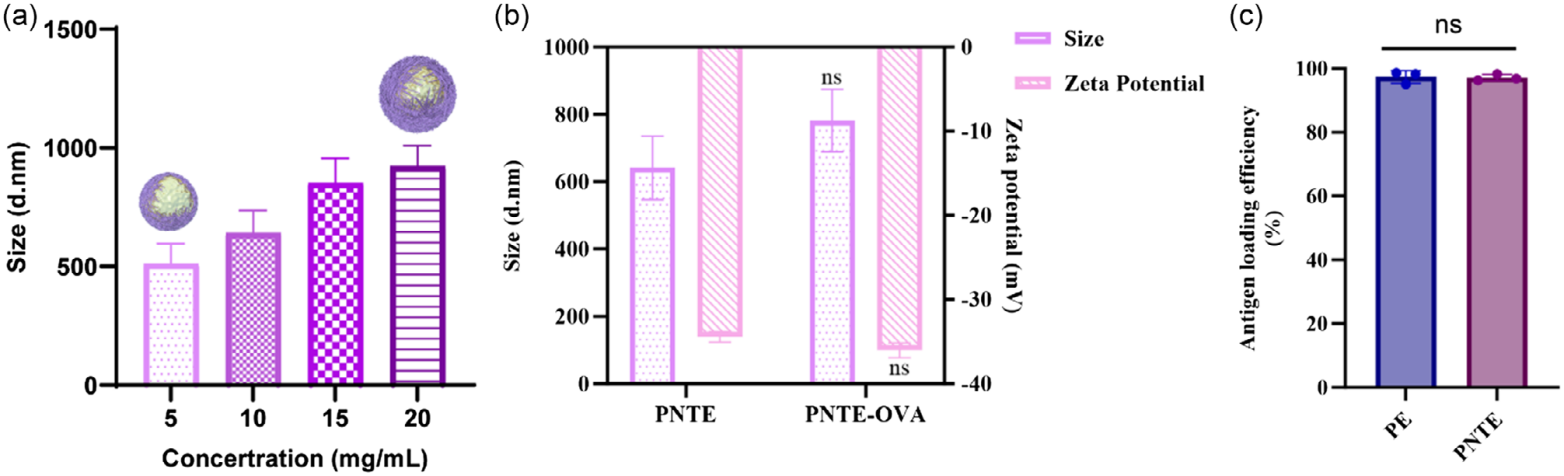
Optimization of PNTEs and their ability to carry antigens. a) The particle size of PNTEs varies with PNT concentration. b) Changes in particle size and potential of PNTEs containing 5 mg·mL^−1^ PNTs after 0.1 mg·mL^−1^ OVA was added (*n* = 3). c) The encapsulation efficiency of Pickering emulsion stabilized with 5 mg·mL^−1^ PNPs and PNTs particles (*n* = 3).

To obtain emulsions with the optimal particle size, four different PNT concentrations were chosen to prepare emulsions, and the results showed that the emulsion particle size of the PNTEs increased with increasing particle concentration (**Figure** **3**a), and only the emulsion containing a low concentration of PNT was stable and could be stored for 14 days (Figure S4, Supporting Information). Small emulsion particle size^[^ ^27^ ^]^ and good stability^[^ ^28^ ^]^ are necessary for vaccine adjuvant to provide a better immune effect, so the particle concentration of Pickering emulsion was set at 5 mg·mL^−1^. This may be because the oil–water interface became saturated with PNT nanoparticles, and some of the particles could not bend to fit the emulsion surface because of the surface tension at the oil–water interface, resulting in an increased emulsion particle size. Additionally, some free particles without a stable emulsion could lead to emulsion aggregation, thus making it unstable.

Successful loading of the antigen onto an emulsion is critical for a Pickering emulsion.^[^ ^29^ ^]^ When 0.1 mg·mL^−1^ ovalbumin (OVA) was loaded onto the PNTEs containing 5 mg·mL^−1^ PNTs, there was a slight change in the particle size and potential of the PNTEs (Figure 3b), with an increase in particle size of ≈140.8 ± 68.97 nm and a decrease in potential of 1.6 ± 0.97 mV. As the amino‐rich antigen is tightly adsorbed within the carboxyl‐rich gaps between the PLGA particles, the amino and carboxyl groups form stable hydrogen bonds to stabilize the loading of the antigen.^[^ ^10^ ^]^ This results in a high‐antigen‐loading rate of PNTE that is similar to that of the PNP‐stabilized Pickering emulsion (PE), both exceeding 90% (Figure 3c). In conclusion, Pickering emulsions stabilized by nonspherical particles were able to be efficiently loaded with antigens.

Based on the preparation of particles and particle‐stabilized Pickering emulsions, the analysis and comparison of adjuvant immune effects will be categorized into two main groups: particles as immune adjuvants and Pickering emulsions as immune adjuvants. For particle‐based adjuvants, the comparison will focus on spherical particles (PNPs) versus nonspherical particles (PMFs, PMTs, and PNTs). For Pickering emulsion‐based adjuvants, the comparison will center on emulsions stabilized by spherical particles (PEs) versus those stabilized by nonspherical particles (PNTEs).

### 2.3 Interaction between Vaccine Adjuvants and Antigen‐Presenting Cells

**Figure 4:**
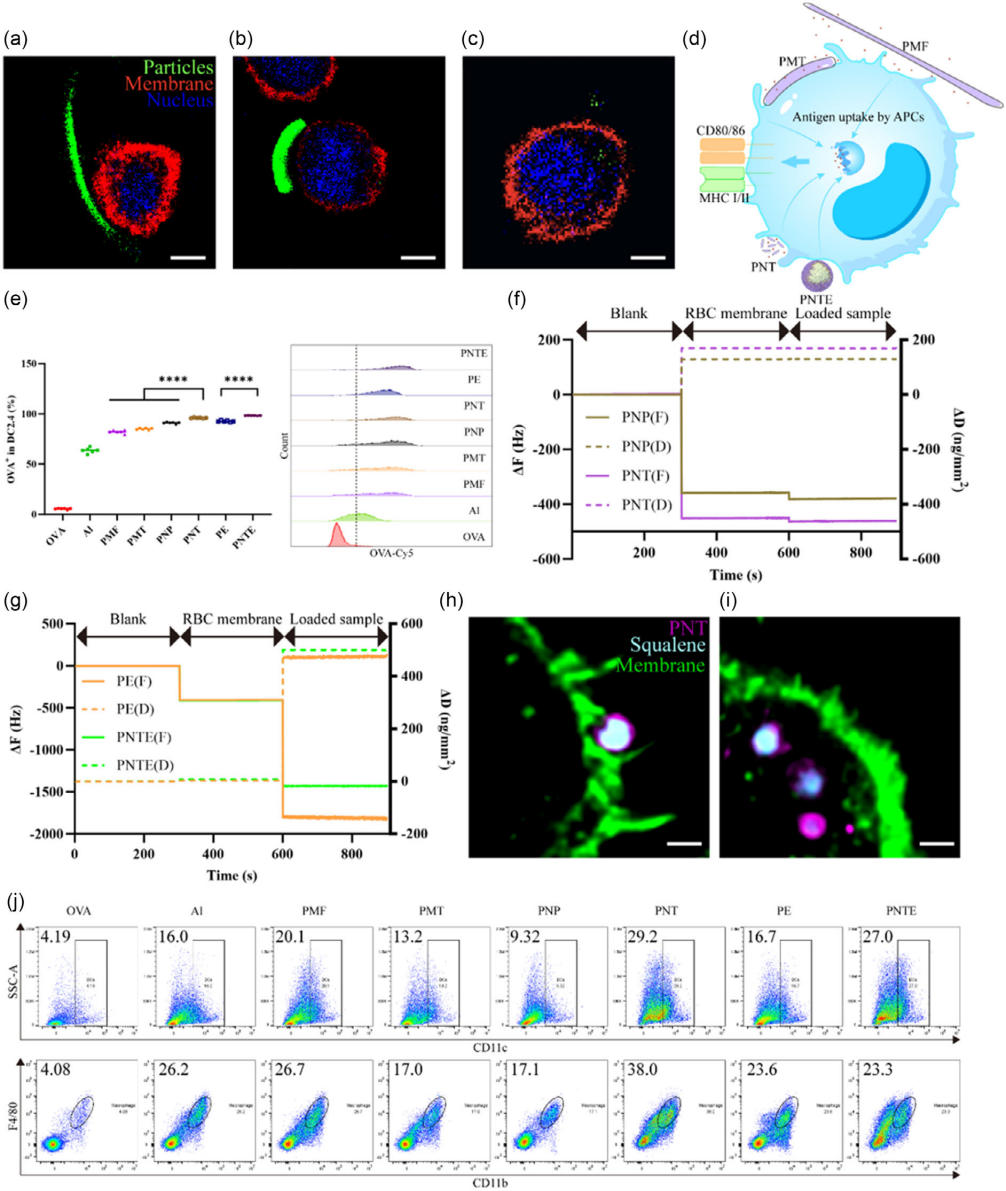
Interaction between vaccine adjuvants and APCs. CLSM images of nonspherical particles: a) PMFs, b) PMTs, and c) PNTs in contact with J774a.1 macrophage. Particles were labeled with Cy5 (green), F‐actin was labeled with phalloidin‐FITC (red), and nuclei were labeled with DAPI (blue) (scale bar = 5 μm). d) Schematic diagram of interaction between adjuvant and APCs. e) Antigen uptake rates of DC2.4 with different antigen‐loaded particles or emulsions (*n* = 6) and its flow cytometry representative diagram. f) QCM‐D analyzed the deformability of particles. g) QCM‐D analyzed the deformability of emulsions. CLSM images of J774a.1 macrophage uptake of PNTEs h) before and i) after, F‐actin was labeled with phalloidin‐FITC (green), PNTs were labeled with Cy5 (purple), and squalene was labeled with Nile red (cyan) (scale bar = 1 μm). j) Representative flow cytometry images of recruited APCs on day 3 by vaccine adjuvants.

The ability of vaccine adjuvants to enhance antigen delivery by antigen‐presenting cells (APCs) is a crucial factor in assessing their immunization effect.^[^ ^30^ ^]^ First, the contact between nonspherical particle adjuvants and macrophages was investigated, and a larger contact area between the adjuvants and APCs was found to be more conducive to the activation of APCs.^[^ ^31^ ^]^ Owing to the long length of PMFs (**Figure** **4**a), when added to a culture medium containing cells, they could act as an extracellular scaffold network, making it easy for cells to adhere to their surface. PMTs (Figure 4b) were more prone to bending owing to the flexibility and anisotropy of the nonspherical PLGA particles, resulting in complete adhesion between the cells and particles. PNTs (Figure 4c) were easily taken up by the cells because of their small particle size. The larger contact area of the nonspherical particles with the APCs facilitated their activation (Figure 4d).

The amount of antigen uptake by APCs indicates the effect of adjuvants.^[^ ^7^ ^]^ Cy5‐labeled OVA was co‐mixed with adjuvants, followed by coculture with DC2.4 cells, a type of APCs, to determine its ability to enhance antigen uptake by APCs. The enhancement of antigen uptake was characterized by flow cytometry (Figure 4e) and was significantly enhanced by the addition of the adjuvant. PNTs had the best effect in promoting antigen endocytosis among all the particles, increasing endocytosis from 5.52% in the pure antigen to 96.02%, which was significantly higher than that of the other three particles. Compared to PEs, PNTEs significantly enhanced antigen endocytosis, reaching up to 98.05%.

Subsequently, to investigate the reason for the enhanced antigen uptake of the emulsions, the deformability of the particles (Figure 4f) and emulsions (Figure 4g) in contact with cell membranes were tested by quartz crystal microbalance with dissipation (QCM‐D). The change in resonant frequency (ΔF) and dissipation (ΔD) when particles or emulsions were in contact with red blood cell membranes was measured using QCM‐D and indicated the flexibility and deformability of the particles or emulsions. As the weight of the QCM‐D chip increases with the addition of a coating, ΔF decreases. Concurrently, ΔD increases if the coating material is flexible and deformable.^[^ ^32^ ^]^ The deformability of the emulsion allowed it to increase the contact area when in contact with APCs (Figure 4h), making it easier for surface antigens to be recognized by APCs. After uptake by the cells, the original shape was restored (Figure 4i).

Adjuvant retention at the injection site creates an environment for immune activation, recruiting more APCs to enhance antigen presentation.^[^ ^30^ ^]^ Therefore, the recruitment of dendritic cells (DCs) and macrophages to the injection site on day 3 was tested. Flow cytometry was used to compare representative images between the pure antigen and vaccine adjuvant (Figure 4j), with PNTs showing significantly enhanced APCs recruitment, recruiting 29.2% DCs and 38.0% macrophages compared to particles. Compared to emulsions, PNTEs recruited macrophages in a manner similar to PEs, but they recruited 10.3% more DCs compared to PEs.

**Figure 5:**
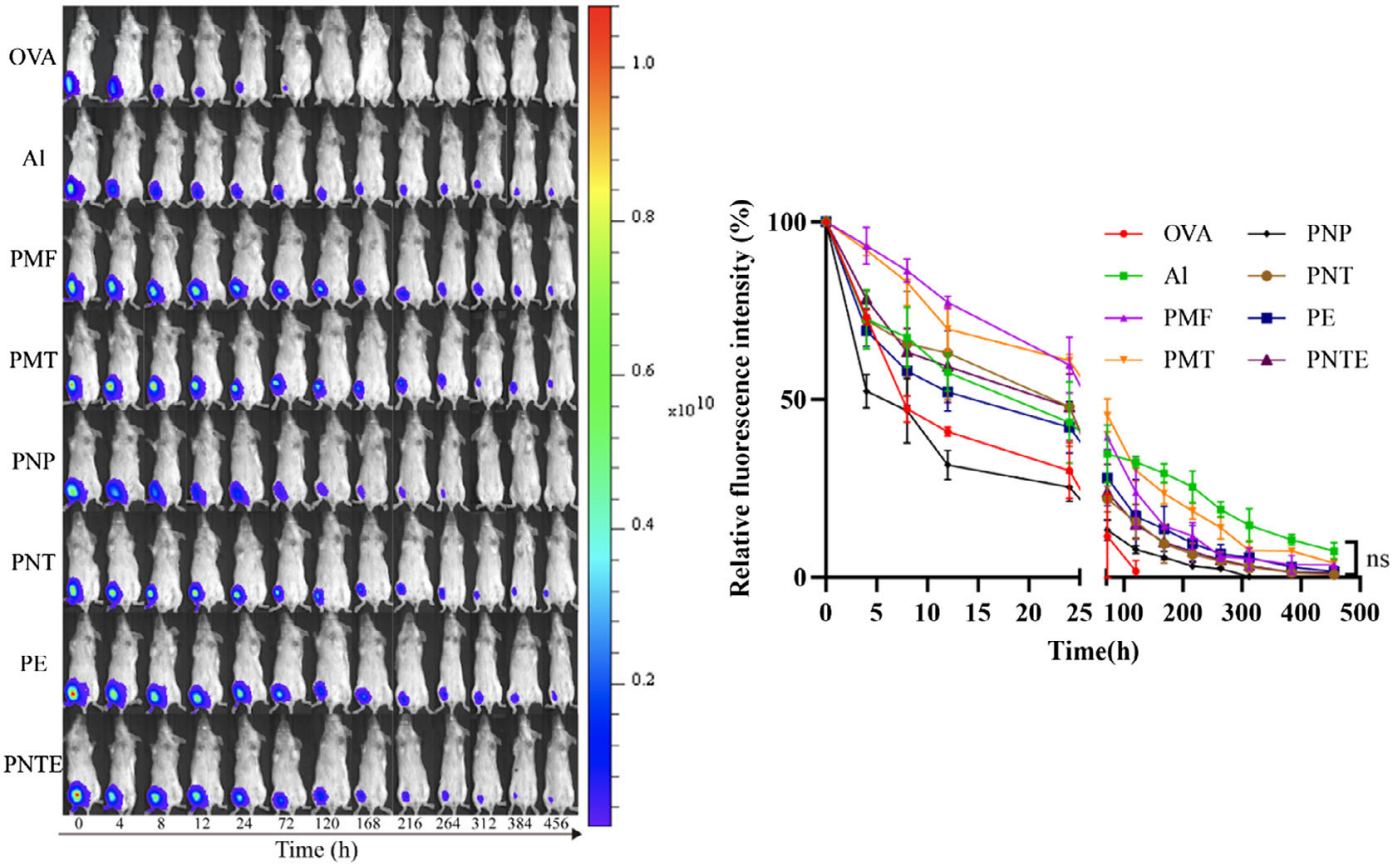
Fluorescence imaging and changes in relative fluorescence intensity of adjuvants at injection site overtime, with antigens labeled with Cy7 (*n* = 3).

Prolonged antigen retention at the injection site allows for extended interaction with APCs.^[^ ^33^ ^]^ To assess the sustained release of the antigen from different dosage forms at the injection site, an adjuvant containing the labeled antigen was injected into the medial thigh muscle of the mice, and antigen retention in each group was continuously observed using an in vivo fluorescence imaging system. Compared to the antigen‐only group, which retained only 10% of the antigen after 72 h, the other groups retained the antigen at the injection site for more than 72 h. The PNP group exhibited a relatively short antigen retention time, retaining only ≈7% at 120 h, whereas the other six groups retained the antigen for more than 456 h. The antigen retention times of the PMFs, PMTs, PNTs, PEs, and PNTEs were similar to those of aluminum (Al) (**Figure** **5**). The addition of the aforementioned adjuvants prolonged the antigen release time and enhanced antigen retention at the injection site.

**Figure 6:**
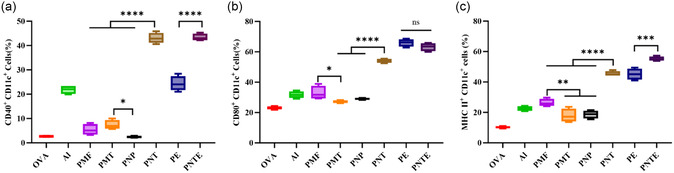
Prolonged in vivo residence time promotes interaction with APCs. Flow cytometry analysis of a) CD40, b) CD80, and c) MHC II expression on BMDCs (*n* = 4).

The ability of an adjuvant to activate bone marrow–derived DCs (BMDCs) is an indicator of its efficacy. The binding of CD40 to the surface of DCs promotes the production of cytokines and chemokines, induces the expression of costimulatory molecules, and promotes the cross‐presentation of antigens.^[^ ^34^ ^]^ The expression levels of CD40 in the PNT group on BMDCs were significantly increased compared to the other particle groups, whereas the levels in the PNTE group were significantly increased compared to the PE group (**Figure** **6**a). CD80, another stimulating factor, binds to the receptor CD28 on the surface of T cells, providing a signal for initial T cell activation and producing a stimulatory effect that promotes T cell activation, proliferation, and differentiation.^[^ ^35^ ^]^ The ability of PNTs to induce CD86 expression within the particle group was significantly higher than that of the other groups, and the induction effect of PMFs was also higher than that of PMTs and PNPs. No significant difference was observed between the PNTE and PE groups (Figure 6b).

After the antigen was taken up by DCs, it was processed via the major histocompatibility complex (MHC) II pathway to form antigen peptides. After binding to intracellular MHC II molecules, it was presented on the surface of DCs and recognized by specific T cells, thereby activating the immune response.^[^ ^36^ ^]^ The expression of MHC II molecules on the surface of DCs indicates the immune‐promoting effects of the adjuvants. The results showed that PNTs and PNTEs had high MHC II expression and that PMFs had higher MHC II expression levels compared to those of PMTs and PNPs (Figure 6c).

### 2.4 Promotion of Humoral and Cellular Immunity

**Figure 7:**
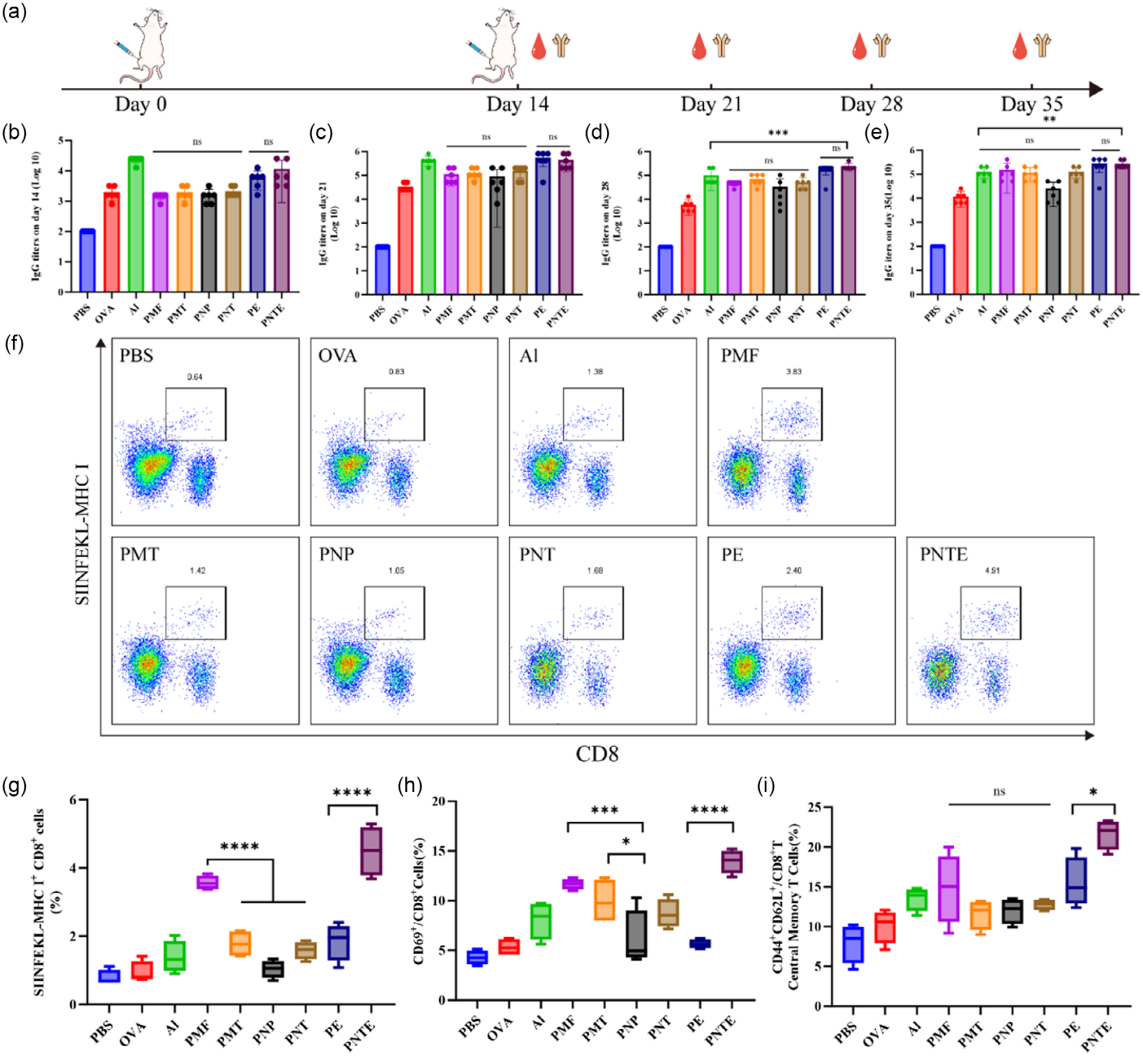
Enhancing effects on humoral and cellular immunity. a) Schematic diagram of the process of immunization. OVA‐specific IgG titers on days b) 14, c) 21, d) 28, and e) 35 (*n* = 6). f) Representative flow cytometry analysis of OVA‐specific cytotoxic T lymphocytes. g) Proportion of OVA‐specific cytotoxic T lymphocytes (*n* = 4). h) Proportion of CD69 in CD8^+^ T cells (*n* = 4). i) Proportion of TCMs (*n* = 4).

Vaccination studies were conducted in mice to evaluate the immune‐enhancing capabilities of the different vaccine adjuvants, with an initial boost administered on day 14 after the first immunization. Mouse serum was collected via orbital blood collection on days 14, 21, 28, and 35, and OVA‐specific IgG titers were measured using enzyme linked immunosorbent assay (**Figure** **7**a). The IgG titer of the Al was higher than that of the other groups on day 14 after the first immunization but gradually lost significance after the first boost. There were no significant differences among the particle groups, and the IgG titer gradually increased and remained stable with the Al after the first boost. The IgG titer of the PNP group was lower than that of the nonspherical particles. Compared to emulsions, there was no significant difference between the PNTE and PE groups; however, the IgG titer of the PNTE group was significantly higher than that of the Al group after day 28 (Figure 7b–e).

In addition to humoral immunity, cellular immunity is a crucial factor for evaluating the efficacy of adjuvants.^[^ ^37^ ^]^ Cytotoxic T lymphocytes are the most relevant functional measure reflecting immune defense acquired through cellular immunity.^[^ ^38^ ^]^ The levels of antigen‐specific cytotoxic T lymphocytes in the spleen cells of immunized mice in each group were detected (Figure 7f). In the particle group, the PMFs increased by 253% compared to the lowest PNP. In the emulsion group, the PNTEs were 265% higher than PEs (Figure 7g). CD69, the earliest molecule expressed during lymphocyte activation,^[^ ^39^ ^]^ was also examined to assess adjuvant efficacy. After immunization, the expression levels of CD69 increased in each adjuvant. The PMFs in the particles increased significantly from 5.33% of OVA to 11.68%, and the PNTEs in the emulsions increased significantly to 13.95% (Figure 7h). The ability to respond to reinfection was also evaluated. Central memory CD8^+^ T cells (TCMs), typically believed to appear after pathogen clearance, can control secondary infections and protect the body from chronic infections and cancer invasion.^[^ ^40^ ^]^ The number of TCMs in each particle group showed no significant differences, whereas that in the PNTE group was 21.65%, which was significantly higher than that in the PE group (15.50%; Figure 7i). Additionally, cytokines play an important role in immune regulation by promoting or inhibiting the activity of specific immune cells, thereby affecting the activity and function of the immune system.^[^ ^41^, ^42^, ^43^ ^]^ An increase in IL‐17A levels (Figure S5, Supporting Information) and low expression of IL‐10 (Figure S6, Supporting Information) indicated that PNTE activated the immune response mediated by T cells and inhibited the production of immunosuppressive effects.

In summary, nonspherical particles effectively extended the residence time at the injection site, yielding superior immune effects compared to spherical particles after 28 days. Among these, PNTs exhibited the most potent activation effect on BMDCs, followed by PMFs, demonstrating a certain level of activation. Notably, PMFs induced the expression of cytotoxic T lymphocytes and early activation of CD8^+^ T cells, which is attributed to the immunostimulatory activity of micrometer‐level PLGA.^[^ ^44^ ^]^ Compared to the emulsion group, there was no significant difference in the immune effects between the PE and PNTE. However, PNTEs had superior effects in promoting BMDC activation and enhancing cellular immunity.

### 2.5 Biosafety and Biocompatibility Assessment

**Figure 8:**
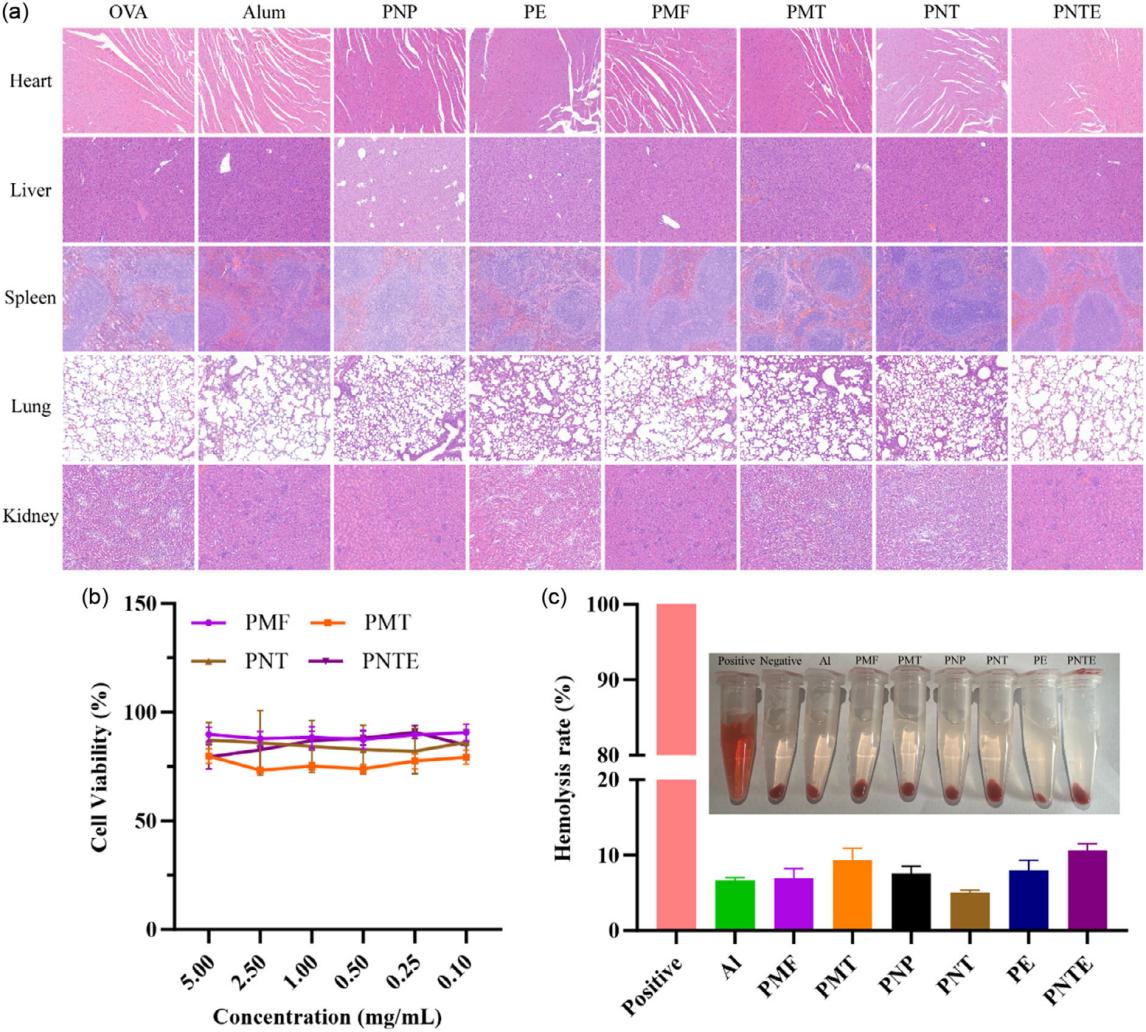
Biosafety and biocompatibility evaluation by a) hematoxylin and eosin staining of vital organ sections from BALB/c mice, b) cell viability of DC 2.4 at different adjuvant concentrations (*n* = 3), and c) hemolysis rate of different adjuvants (*n* = 3).

Ensuring the safety of adjuvants is of paramount importance for their development.^[^ ^45^ ^]^ In the present study, we selected commercial Al adjuvants as a control group and thoroughly investigated the safety of all experimental groups by considering system toxicity, tissue sectioning, cell viability, and hemolysis rate. There were no significant differences in the biochemical indices of the mice after normal immunization, and there was no obvious nephrotoxicity or hepatotoxicity in any group (**Table** **1**). Notably, on day 28 post‐immunization, examination of vital organs, including the heart, liver, spleen, lungs, and kidneys, revealed no significant pathological changes (**Figure** **8**a). Furthermore, variations in the adjuvant concentration had no discernible impact on cell viability (Figure 8b) and did not lead to hemolysis (Figure 8c). In summary, the PMFs, PMTs, PNTs, and PNTEs demonstrated excellent biosafety and biocompatibility.

**Table 1:** Biochemical analyses of formulations.

	ALP [U L^−1^]	LDH [U L^−1^]	AST [U L^−1^]	ALT [U L^−1^]	BUN [mmol L^−1^]
PBS	30 ± 3.7	368 ± 89.4	60 ± 15.8	20 ± 5.7	3.8 ± 0.4
OVA	31 ± 3.8	295 ± 102.7	49 ± 7.4	19 ± 6.8	4.0 ± 0.5
Al	37 ± 7.7	326 ± 74.7	63 ± 8.5	17 ± 4.8	3.7 ± 0.3
PMF	40 ± 5.9	343 ± 89.9	63 ± 7.2	27 ± 8.6	5.0 ± 0.9
PMT	37 ± 6.5	248 ± 85.9	58 ± 5.9	24 ± 5.0	5.3 ± 0.9
PNP	39 ± 2.9	352 ± 50.6	60 ± 7.5	30 ± 19.2	4.2 ± 0.4
PNT	32 ± 2.7	297 ± 102.6	56 ± 8.2	23 ± 5.9	4.8 ± 0.6
PE	35 ± 5.3	302 ± 68.0	49 ± 6.4	19 ± 6.5	4.4 ± 0.8
PNTE	36 ± 13.4	265 ± 92.6	55 ± 11.5	24 ± 4.0	3.9 ± 0.5

Abbreviations: ALP, alkaline phosphatase; LDH, lactate dehydrogenase; AST, aspartate transaminase; ALT, alanine transaminase; BUN, blood urea nitrogen.

## 3 Conclusion

In this study, PMF, PMT, and PNT particles with different lengths and sizes were successfully prepared. Because of the increased contact area with APCs, nonspherical particles have an enhanced antigen reservoir effect and can recruit more APCs, which is better than spherical particles for both humoral and cellular immunity. Notably, nanoscale nonspherical particles significantly enhanced the antigen uptake and activation of BMDCs. However, in the composite adjuvant, the interaction between nonspherical particles and other materials, and the changes in the layout, may have an impact on the immune effect. Therefore, the Pickering emulsion stabilized by nonspherical particles was also evaluated for the first time as an immune adjuvant. In the composite adjuvant, the Pickering emulsion stabilized by nonspherical particles provided improved flexibility, resulting in a larger contact area with APCs, excellent cellular immune enhancement, and overall excellent humoral immune effects. The nonspherical particles, with their distinct geometric and surface properties, can further improve the immunogenicity of Pickering emulsions, offering a novel approach to vaccine development that leverages the advantages of particle shape diversity. We also conducted a preliminary evaluation of the biosafety and biocompatibility of the adjuvants, confirming their advantages in these fields. Nonspherical nanoparticles show great potential as adjuvant candidates, whether used as particles or emulsion adjuvants. With progress in fluid mechanics, microfluidic control, nanotechnology, and other related fields, the preparation technology of nonspherical particles will become more refined and diversified. In addition, the preferential use of environmentally friendly and sustainable materials, such as natural polymers and biodegradable substances, contributes to health and environmental protection. For stable emulsions with nonspherical particles, it is important to improve the emulsion stability and performance. By combining the unique properties of nonspherical particles and Pickering emulsions, an optimized design scheme can intelligently and effectively use emulsion adjuvants.

## 4 Experimental Section

4.1

4.1.1

##### Materials

PLGA (lactic acid/glycolic acid, 75:25 [w/w], *M*_w_ ≈ 13 k Da) was purchased from Jinan Daigang Biomaterial Co., Ltd. (Shandong, China). Polyvinyl alcohol 217 was purchased from Kuraray Co., Ltd. (Japan). Dichloromethane (CH_2_Cl_2_) and ethyl acetate (C_4_H_8_O_2_) were purchased from Aladdin (Shanghai, China). Ammonium acetate (CH_3_COONH_4_) was purchased from Sinopharm Chemical Reagent Co., Ltd. Model antigen OVA, bovine serum albumin (BSA), and squalene were purchased from Sigma Aldrich (USA).

Phalloidin‐fluorescein isothiocyanate (FITC) and Nile red were purchased from Solarbio Biological Technology Co., Ltd. (China). Cy5 mono‐reactive N‐hydroxysuccinimide esters was purchased from Fanbo Biochemicals (China). Horseradish peroxidase (HRP)‐labeled goat anti‐mouse IgG was purchased from Abcam. Micro‐bicinchoninic acid (BCA) assay kits were purchased from Thermo Fisher Scientific. Fluorescent‐labeled CD11c, CD11b, CD8, CD40, CD44, CD62L, CD80, CD86, *SIINFEKL* MHC I, MHC II, and F4/80 monoclonal antibodies were purchased from eBioscience, Inc. Ghost Dye v510 was purchased from eBioscience.

The complete medium for culturing DCs, macrophages, and splenocyte cells was the Roswell Park Memorial Institute (RPMI) 1640 or Dulbecco's Modified Eagle's Medium (DMEM) medium supplemented with 10% (v/v) fetal bovine serum and 1% (v/v) penicillin–streptomycin were all purchased from Gibco. Phosphate‐buffered saline (PBS) and PBS/Tween 20 (PBST) were purchased from Solarbio. The cell counting kit‐8 was purchased from Donjindo (Japan).

##### Mice

Female Bagg Albino Laboratory‐bred/c (BALB/c) mice (6–8 weeks old) and male C57BL/6 mice (6–8 weeks old) were obtained from Sinogenetic Biotechnology Co., Ltd (Beijing, China). All experiments were strictly conducted in accordance with the National Research Council's Guide for the Care and Use of Laboratory Animals and performed in strict accordance with the Regulations for the Care and Use of Laboratory Animals and Guideline for Ethical Review of Animals (China, GB/T 35892‐2018). All animal experiments were reviewed and approved by the Animal Ethics Committee of the Institute of Process Engineering (approval ID: IPEAECA2023054).

##### Preparation of Particles and Emulsions: Preparation of PMFs

An amount of 30 mg of PLGA was dissolved in 1 mL of ethyl acetate to prepare the oil phase. And, 750 mg of ammonium acetate was dissolved in 75 mL of deionized water containing 1.5 wt% PVA217 to create the aqueous phase. Gradually the oil phase was added to the aqueous phase while stirring at 600 rpm. Stirring was continued for 4 h to allow complete extraction of ethyl acetate by the water, which induced the precipitation of PLGA and the formation of microfibers. The microfibers were washed three times with deionized water and then centrifuged at 14 000 rpm for 10 min. The precipitate was resuspended in deionized water and freeze‐dried the suspension to obtain PMF powder, which was suitable for storage and subsequent use.

##### Preparation of Particles and Emulsions: Preparation of PMTs

An amount of 10 mg of freeze‐dried PMF powder was weighed and it was dispersed in 2 mL of PBS to achieve a concentration of 5 mg·mL^−^^1^. This suspension was subjected to ultrasound treatment using a Digital Sonifier 250 (Branson Ultrasonics Corporation) with an on 4s/off 4s program at 30% power for 2 min. This process resulted in a 2 mL PBS solution containing PMTs at a concentration of 5 mg·mL^−1^.

##### Preparation of Particles and Emulsions: Preparation of PNPs

An amount of 30 mg of PLGA was dissolved in 1 mL of dichloromethane to form the oil phase. The aqueous phase was prepared by mixing 5 mL of deionized water containing 1.5 wt% PVA217. The oil and aqueous phases were combined in a 50 mL centrifuge tube and sonicated using a cell crusher at 30% power (on 4s/off 4s) for 3 min to create an oil‐in‐water emulsion. This emulsion was stirred at 600 rpm for 4 h to fully volatilize the dichloromethane, which resulted in the formation of PNPs. The nanoparticles was washed three times with deionized water, centrifuged at 14 000 rpm for 10 min, and resuspended in deionized water. The suspension was freeze‐dried to obtain PNP powder for storage and future experiments.

##### Preparation of Particles and Emulsions: Preparation of PNTs

An amount of 30 mg of PLGA was dissolved in 6 mL of dichloromethane to create the oil phase. In a separate container, 500 mg of ammonium acetate was dissolved in 50 mL of deionized water containing 1.5 wt% PVA217 to form the aqueous phase. The oil phase was mixed with the aqueous phase using a homogenizer at a high speed (setting 6) for 2 min. The mixture was transferred to a magnetic stirrer and stirring was continued at 600 rpm for 4 h to fully volatilize the dichloromethane, resulting in the formation of PNTs. The PNTs was washed three times with deionized water, centrifuged at 20 000 rpm for 10 min, and resuspended in deionized water. The suspension was freeze‐dried to obtain PNT powder for easy storage and subsequent use.

##### Preparation of Particles and Emulsions: Preparation of PMFE/PMTE/PNPE/PNTE Pickering Emulsions

An amount of 5 mg of freeze‐dried PMF, PMT, PNP, or PNT powder was dispersed in 0.9 mL of PBS to form the aqueous phase. And, 0.1 mL of squalene was added as the oil phase. The two phases were mixed and sonicated using a cell crusher at 20% power (on 4s/off 4s) for 1 min to prepare the particle‐stabilized Pickering emulsion.

##### Characterization of Particles and Emulsions

The average sizes of emulsions and zeta potential were measured by a Zetasizer Nano ZS (Malvern, UK). The average sizes of particles were measured by Nano Measurer 1.2 software. The surface morphology of the particles was observed by a JSM‐6700 SEM (JEOL, Japan). The surface morphology of the emulsions was observed by a Regulus8100 Cryo‐SEM (Hitachi, Japan). Fluorescence images were obtained through ECLIPSE Ti2 CLSM (Nikon, Japan).

##### Vaccine Preparation and Characterization: Preparation of Pure Particles Vaccines

The OVA antigen and particles aqueous solution were mixed to fabricate pure particle vaccines, the final concentration of OVA was 100 μg·mL^−1^, and the concentration of each particle was 5 mg·mL^−1^.

##### Vaccine Preparation and Characterization: Preparation of Pickering Emulsion Vaccines

The OVA antigen and PE/PNTE solution were mixed to fabricate Pickering emulsion vaccines, the final concentration of OVA was 100 μg·mL^−1^, and the concentration of each particle was 5 mg·mL^−1^.

##### Vaccine Preparation and Characterization: Antigen Loading Rate Calculation

The antigen load rate of each vaccine formulation was calculated by measuring the free antigen in the external quantitative aqueous phase using the dialysis method. Free antigen was separated from the encapsulated emulsion by dialysis in PBS buffer solution at 4 °C using Spectra/ForFloat‐A‐Lyzer G2 dialysis system (*M*_w_ ≈ 300 k Da) until the amount of antigen was basically unchanged. At the same time, dialysis of blank particles and emulsions without OVA load was performed in PBS buffer solution as a control to remove the background related to the adjuvant. Antigens in PBS buffer solution were detected using a microBCA protein assay kit. The antigen loading rate of each vaccine formulation could be calculated using the following equation:

(1) $$\text{Antigen loading rate }\left(\%\right)=\frac{\text{Total antigens}-\text{free antigens in PBS}}{\text{Total antigens}}\times 100\%$$

##### Vaccination Protocols

Female BALB/c mice (6–8 weeks old, *n* = 6 per group) were immunized twice, on days 0 and 14, by injecting 100 μL of the vaccine into the hind leg muscle. The groups of animals and their corresponding vaccine formulations are presented in **Table** **2**.

**Table 2:** The groups of animal immunization and corresponding vaccine formulations.

Group classification	Group name	Particle content	V (oil):V (water)	OVA content [μg]
Blank group	PBS	–	–	–
Negative controls	OVA	–	–	10
Positive controls	Aluminum	–	–	10
Experimental group	PNP	500 μg	–	10
	PMF	500 μg	–	10
	PMT	500 μg	–	10
	PNT	500 μg	–	10
	PE	500 μg	1:9	10
	PNTE	500 μg	1:9	10

##### Recruitment of APCs

Immunization was performed with 100 μL of each vaccine. On day 3, muscle tissue from the injection site was collected to prepare a cell suspension. The cell suspension was centrifuged, and the supernatant was discarded. The cells were then washed with PBS and stained with 100 μL of a staining buffer containing antibodies: BV510‐Ghost dye, BV605‐CD11C, BV421‐CD11b, and FITC‐F4/80. After staining, the cells were washed again with staining buffer and resuspended. Data was processed using flow cytometry (Cytoflex, Beckman, USA) and analyzed with FlowJo 10.8.1 software (Becton, Dickinson & Company, USA), with flow cytometry acquiring data from over 10 000 target cells for analysis.

##### Quartz Crystal Microbalance with Dissipation

The deformability of particles and emulsions was measured by Q‐SENSE E4 (Biolin Scientific, Sweden). First, a stable baseline was established via the blank chip (QSX301). Then, the red blood cell membrane and particles/emulsions were sequentially coated onto the blank chip at a rotational speed of 4000 rpm·min^−^^1^ and detected using QCM‐D. The extraction of the red blood cell membrane was carried out according to the methods in ref. [46].

##### Enzyme‐Linked Immunosorbent Assay for Detecting Antigen‐Specific Antibodies

On days 14, 21, 28, and 35 after the first vaccination, blood was collected from the orbital veins of mice, and serum was separated to measure antibody levels. First, 96‐well high‐binding plates were coated overnight at 4 °C with 100 μL of coating buffer (50 mM Na_2_CO_3_–NaHCO_3_, pH 9.6) containing OVA (20 μg·mL^−^^1^). The following day, the plates were washed three times with wash buffer (PBST, 0.05% Tween 20 in PBS) and blocked for 1 h at 37 °C with blocking buffer (0.5% BSA in PBST). Next, serial dilutions of serum were added to the washed plates and incubated at 37 °C for 45 min. The plates were then incubated with 100 μL of horseradish peroxidase‐conjugated IgG antibody (1:50 000 dilution) in dilution buffer (0.1% BSA in PBST) for 40 min at 37 °C. After six washes, 100 μL of 3,3′,5,5′‐tetramethylbenzidine substrate was added to each well, and color development was allowed for 20 min. The reaction was stopped with 50 μL of 1 M H_2_SO_4_, and the optical density at 450 nm was measured. Antibody titers were calculated as the serum dilution corresponding to twice the optical density.

##### Cell Activation in BMDCs Essay

BMDCs (1 × 10^6^ cells·mL^−1^) were isolated from bone marrow of male C57BL/6 mice at 6–8 weeks of age and inoculated into 24‐well culture plates, and 1 mL of RPMI 1640 complete medium containing stimulatory factors (10 ng·mL^−1^ granulocyte‐macrophage colony‐stimulating factor and 20 ng·mL^−1^ interleukin‐4) was added to each well. The plates were incubated at 37 °C in an incubator with 5% CO_2_ changed every other day for 6 days and then removed for experiments.

Activation of BMDCs: BMDCs were incubated with different vaccine preparations (not fluorescently labeled with OVA) for 12 h. BMDCs were stained with BV510‐Ghost dye, PerCP‐Cy5.5‐CD11c, APC‐CD40, PC7‐CD80, and V450‐MHC II. The cell rates of CD40^+^CD11c^+^, CD80^+^CD11c^+^, and MHC II^+^CD11c^+^ were detected by flow cytometry.

Flow cytometry obtained over 8000 target cells for analysis and FlowJo 10.8.1 software (Becton, Dickinson & Company, USA) were used for data processing.

##### Hemolysis of Antigen–Adjuvants

To analyze the hemolytic effect of antigen–adjuvants, 100 μL of injection concentration of antigen–adjuvants was added to 1 mL of PBS solution containing 20 μL of mouse red blood cells. The mixture was incubated at 37 °C for 2 h. After incubation, the solution was centrifuged at 3000 rpm for 15 min. Photos were taken, and absorbance was measured at 540 nm, with a reference wavelength of 630 nm. Deionized water served as the positive control group, while pure PBS was the negative control group. The hemolysis rate was calculated using the following formula:

(2) $$\text{Hemolysis ratio }\left(\%\right)\;=\;\frac{\text{OD}\left(\text{sample}\right)\text{ − OD(negative)}}{\text{OD}\left(\text{positive}\right)\text{ − OD(negative)}}\text{ × 100\%}$$

##### Cell Line

Monocyte macrophage J774A.1 cell line derived from BABL/c mice and DC2.4 cell line derived from mouse BMDCs were purchased from Procell Life Science & Technology Co., Ltd. (Wuhan, China).

##### Cellular Uptake Essay

An amount of 100 μL of DMEM complete medium containing 10^4^ cells·mL^−1^ J774A.1 was inoculated in the center of a 35 mm glass‐bottomed Petri dish and incubated in an incubator (37 °C, 5% CO_2_) for 3 h to make it adherent to the wall, after which 30 μL PNTE (Cy5‐labeled PNT, Nile red‐labeled squalene) was added and continue to incubate in the incubator for 4 h. Finally, fibrous actin (F‐actin) was labeled with phalloidin‐FITC and observed by CLSM.

##### Antigen Uptake Essay

Antigen uptake by DC2.4 cells:DC2.4 cells were incubated with different vaccine preparations (Cy5‐labeled OVA) for 12 h. The cell rate of OVA^+^ was detected by flow cytometry. Flow cytometry obtained a total of 20 000 cells for analysis and FlowJo 10.8.1 software (Becton, Dickinson & Company, USA) was used for data processing.

##### Cell Viability Detection

First, a cell suspension containing 10^3^ DC2.4 cells (100 μL/well) was inoculated in a 96‐well plate. The plates were precultured in an incubator (at 37 °C, 5% CO_2_). Afterward, 30 μL of the injected concentration sample was added to each well and incubation was continued in the incubator for 12 h. The medium was then removed and 110 μL of medium containing 10 μL of Cell Counting Kit‐8 (CCK8, Dojindo) solution was added to each well. The plates were incubated in the incubator for 1–4 h. Finally, the absorbance at 450 nm was measured using an enzyme meter.

##### Humoral and Cellular Immune Response

Mice were humanely executed on day 35 after immunization, and the spleens were dissected on an ultraclean bench to prepare the spleen cell suspension. The spleen cell suspension was diluted to 2 × 10^6^ cells·mL^−1^, and 500 μL of spleen cell suspension and 500 μL of RPMI 1640 complete medium (containing 8 μg·mL^−1^ OVA) were added to a 24‐well plate and incubated in a cell culture incubator at 37 °C for 60 h. After centrifugation, the cells at the bottom were divided into three equal parts.

Detection of Cytotoxic T Lymphocytes: The proportion of SIINFEKL MHC I^+^CD8^+^ cells in splenocytes was detected by flow cytometry after staining with monoclonal antibodies (flexibility dye‐DAPI, V450‐CD3, FITC‐CD8, and PE‐Cy7‐SIINFEKL MHC I) in flow cytometry buffer.

Detection of Early T‐Cell Activation: The proportion of CD69^+^CD8^+^ cells in splenocytes was detected by flow cytometry after staining with monoclonal antibodies (flexibility dye‐DAPI, V450‐CD3, FITC‐CD8, and APC‐Cy7‐CD69) in flow cytometry buffer.

Detection of Central Memory T Cells: The proportion of CD44^+^CD62L^+^/CD8^+^ cells in splenocytes was detected by flow cytometry after staining with monoclonal antibodies (flexibility dye‐DAPI, V450‐CD3, FITC‐CD8, APC‐CD44, and PE‐CD62L) in flow cytometry buffer.

Flow cytometry obtained over 10 000 target cells for analysis and FlowJo 10.8.1 software (Becton, Dickinson & Company, USA) were used for data processing.

##### Tissue Section Experiments

A total of 200 μL of vaccine of the same formulation as immunization was administered by 2 intramuscular injections on days 0 and 14. Sections of the hearts, livers, spleens, kidneys, and lungs of mice were stained on day 28 to detect pathological changes in each organ.

##### Statistical Analysis

All statistical analyses were performed using GraphPad Prism 10.1.2 software (GraphPad Software, USA). Results were expressed as mean ± s.e.m. Data were analyzed by one‐way analysis of variance with Tukey's post hoc test. Significant differences between the groups were expressed as *P < 0.05, **P < 0.01, ***P < 0.001, and ****P < 0.0001.

## Conflict of Interest

The authors declare no conflict of interest.

## Author Contributions

**Yanan Li**: conceptualization (lead); data curation (lead); formal analysis (lead); investigation (lead); methodology (lead); software (lead); visualization (lead); writing—original draft (lead); writing—review & editing (lead). **Jie Teng**: data curation (equal); formal analysis (equal); investigation (equal); methodology (equal); validation (equal). **Qiuting Chen**: data curation (equal); investigation (equal). **Qingze Fan**: methodology (equal). **Hiroyuki Oku**: methodology (supporting); resources (supporting); writing—review & editing (supporting). **Guanghui Ma**: funding acquisition (supporting); project administration (supporting); resources (supporting); supervision (supporting); writing—review & editing (supporting). **Jie Wu**: funding acquisition (supporting); investigation (supporting); methodology (supporting); project administration (supporting); resources (supporting); supervision (supporting); writing—review & editing (supporting)

## Supporting information

Supplementary Material

## Data Availability

Research data are not shared.
