# Supplementary material for: The Importance of Particle Shape: Effect of Nonspherical Particles and Their Stabilized Pickering Emulsions on Immunization Efficacy
Source: Small Sci. 2025 Apr 20;5(6):2400527. doi: 10.1002/smsc.202400527 (PMC12168590; doi:10.1002/smsc.202400527)
Supplement: Supplementary file 1 — Supplementary Material [file SMSC-5-2400527-s001.pdf]

Supporting Information:

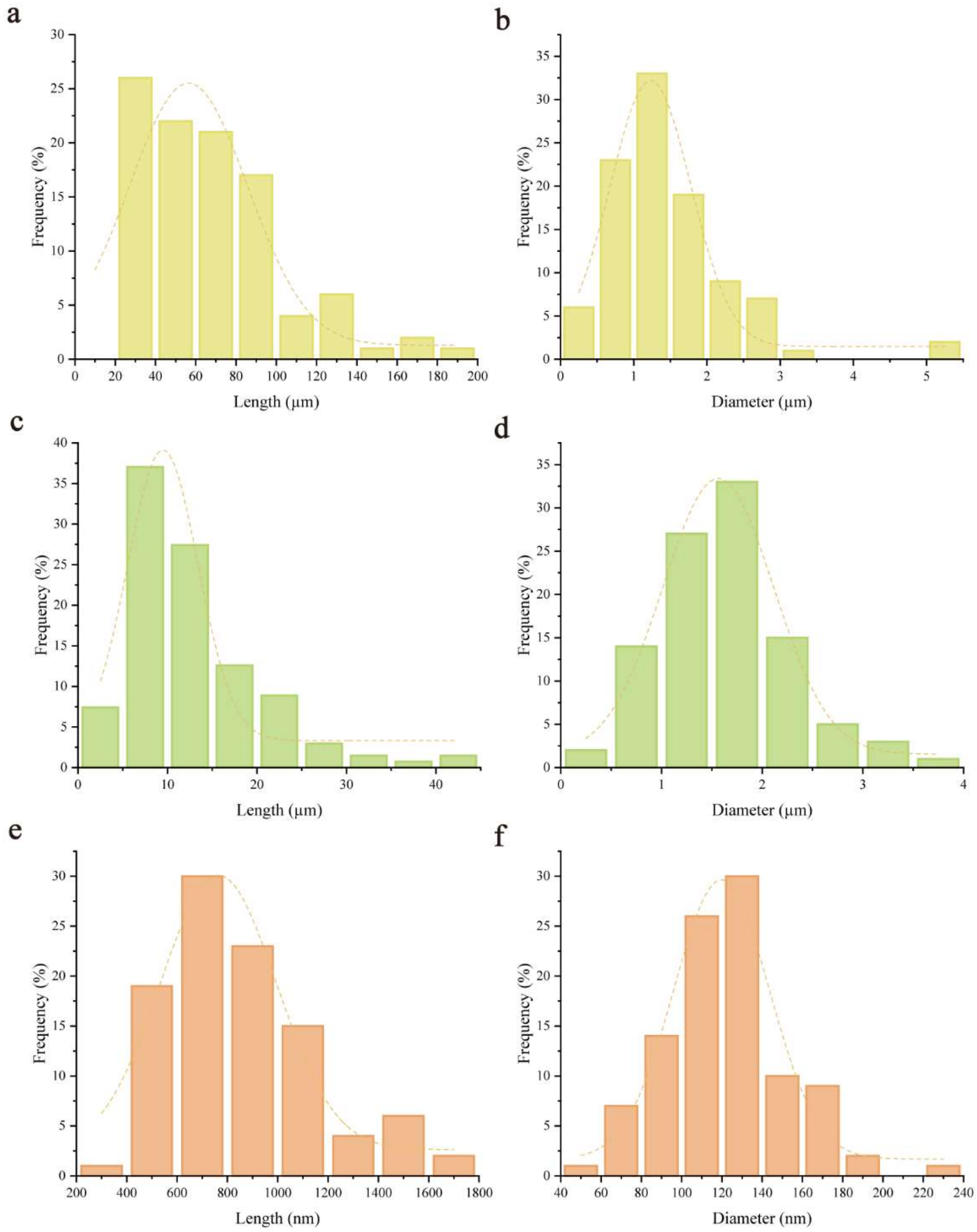

Figure S1. (a) Length distribution and (b) diameter distribution of PMF; (c) Length distribution and (d) diameter distribution of PMT; (e) Length distribution and (f) diameter distribution of PNT.

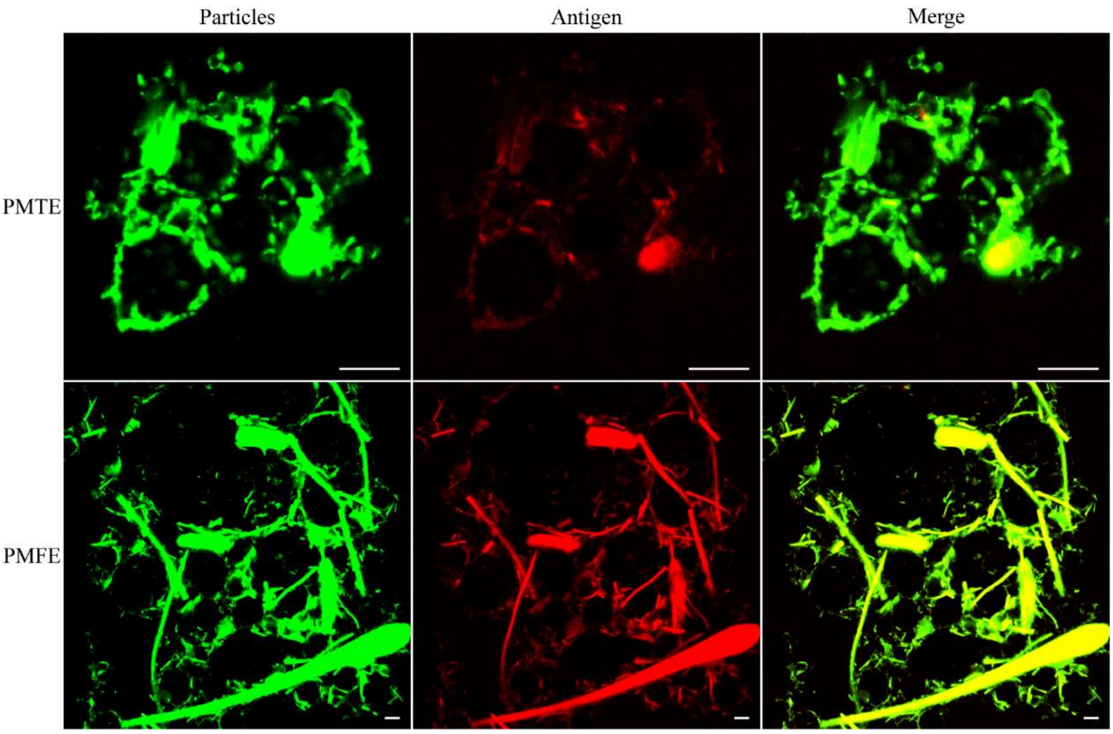

Figure S2. CLSM images of PMT-stabilized emulsion (PMTE) and PMF-stabilized emulsion (PMFE), scale bars are 10μm.

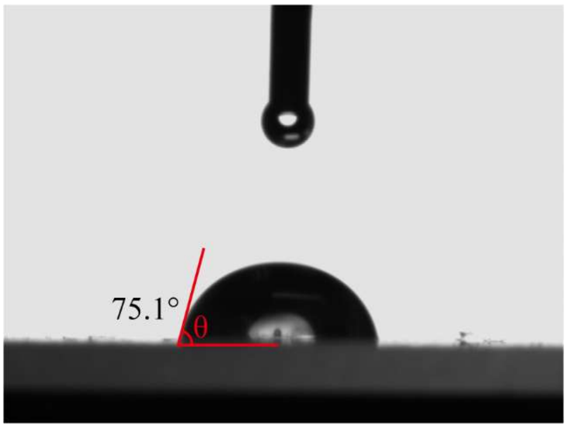

Figure S3. The contact angle between PNTs and water.

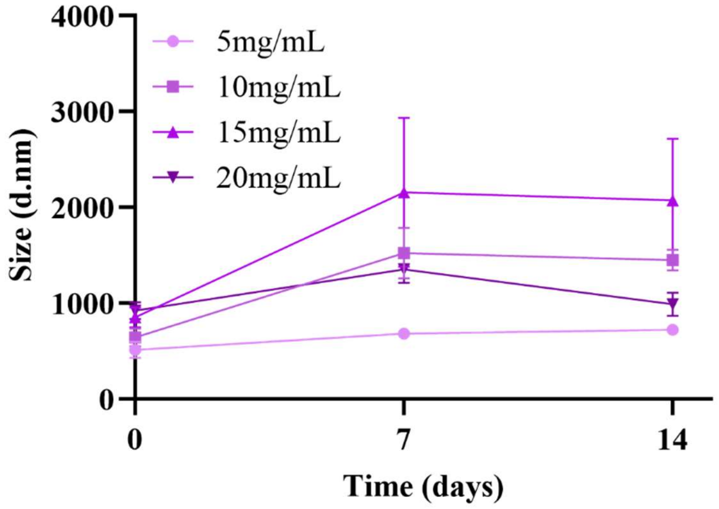

Figure S4. Variation of particle size of PNT stabilized Pickering emulsion at different concentrations with time.

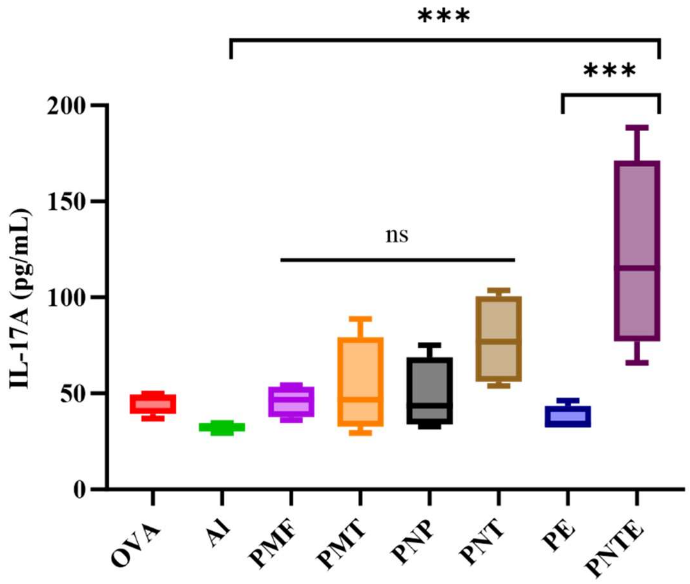

Figure S5. Expression of IL-17A in the supernatant of splenocytes after antigen stimulation. (n=4)

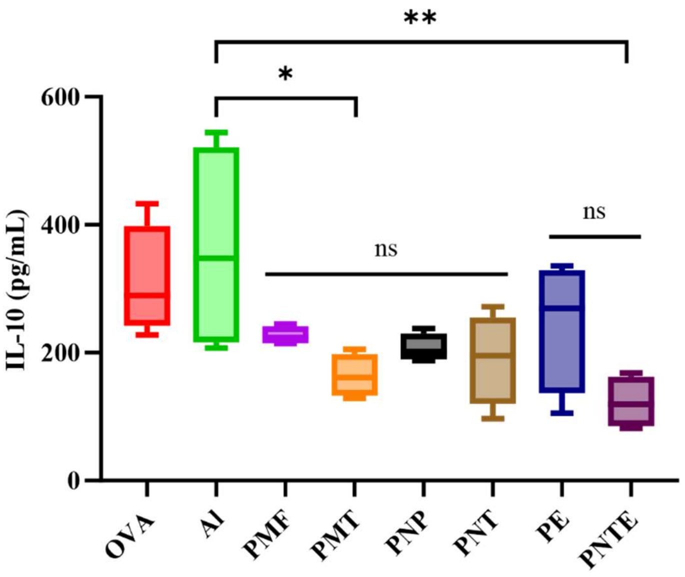

Figure S6. Expression of IL-10 in the supernatant of splenocytes after antigen stimulation. (n=4)
